# Fungal Taxonomy, Phylogeny, and Ecology: A Themed Issue Dedicated to Academician Wen-Ying Zhuang

**DOI:** 10.3390/jof8121294

**Published:** 2022-12-11

**Authors:** Cheng Gao, Lei Cai

**Affiliations:** State Key Laboratory of Mycology, Institute of Microbiology, Chinese Academy of Sciences, Beijing 100101, China

We are honored and privileged to edit this Special Issue, “Fungal Taxonomy, Phylogeny, and Ecology: A Themed Issue Dedicated to Academician Wen-Ying Zhuang”.

Professor Wen-Ying Zhuang is an outstanding mycologist in China and worldwide. Over the past 46 years, she has comprehensively investigated fungal biodiversity in forests, deserts, and plateaus habitats from 26 provinces and districts of China and accumulated numerous priceless resources for scientific research and economic utilization. She has discovered and described 1 new family, 13 new genera, and 360 new species of fungi, and resolved numerous taxonomic and nomenclature problems. Her collaboration with international colleagues contributed greatly to the selection of fungal DNA barcodes and the phylogenetic reconstruction of Leotiomycetes, Helotiales, and Hypocreales. She is the senior author of 280 articles, editor and co-editor of 18 monographs, independent editor of worldwide monographs of 3 important fungal genera, co-editor of Dictionary of the Fungi (v9), and co-Editor-in-Chief of Flora Sporophytae Sinicae. Prof. Wen-Ying Zhuang has been elected as a CAS academician, TWAS academician, IMA executive committee member, and MSA fellow. In honor of her outstanding contribution, one fungal genus *Wenyingia* and one bacterial genus *Wenyingzhuangia* have been named after her. More recently, her work on *Trichoderma* has renewed our understanding of its biodiversity and helped discover many potentially highly capable strains with significant enzyme profiles in degrading agricultural wastes.

In this Special Issue, we are pleased to publish a comprehensive assemblage of 23 papers covering fungal taxonomy, phylogeny, and ecology, in which 76 new taxa from a broad taxonomic group and different ecological habitats are reported.

Prof Wenying Zhuang and colleagues reported three new species of *Clonostachys* (Hypocreales, Ascomycota) from China, namely, *Clonostachys chongqingensis* sp. nov., *Clonostachys leptoderma* sp. nov., and *Clonostachys oligospora* sp. nov. [1]. Using contaminated substrates of edible fungi from North China, Cao et al. [2] detected 10 *Trichoderma* species, including 3 new species in Harzianum clade, *T. auriculariae* sp. nov., *T. miyunense* sp. nov., and *T. pholiotae* sp. nov. From the green mold diseased fruitbody of *Ganoderma sichuanense*, An et al. [3] described *Trichoderma ganodermatigerum* as a new species and reported *Trichoderma koningiopsis* as a new fungal pathogen on *Ganoderma sichuanense* fruitbodies. From 526 strains isolated from diseased watermelon in 8 growing provinces in China, Guo et al. [4] detected 12 known species of *Colletotrichum*, with *Colletotrichum kaifengense* and *Colletotrichum magnum* being the most aggressive to watermelon. Focusing on the large-spored *Alternaria* associated with Compositae plants in China, Zhao et al. [5] discovered five new species, namely, *Alternaria anhuiensis*, *A. coreopsidis*, *A. nanningensis*, *A. neimengguensis*, and *A. sulphureus*. From 353 *Calonectria* strains isolated from leaf blight pathogen of *Eucalyptus* plantations and adjacent plantings, as well as natural forests, Liu et al. [6] identified six known *Calonectria* taxa and one new species, *Calonectria minensis*. In a similar study, Zhang et al. [7] identified five new species, namely, *Calonectria cassiae*, *C. guangdongensis*, *C. melaleucae*, *C. shaoguanensis,* and *C. strelitziae*, based on isolates from leaf spots, stem blights, and root rots of species of *Arachis*, *Cassia*, *Callistemon*, *Eucalyptus*, *Heliconia*, *Melaleuca,* and *Strelitzia* plants in Guangdong province. Relying on newly collected *Otidea* specimens from northern China and herbarium specimens deposited in three important Chinese fungus herbaria (HMAS, HKAS, HMJAU), Xu et al. [8] recognized 16 species of *Otidea* in China, of which 7 new species were described, namely, *Otidea aspera*, *O. cupulata*, *O. filiformis*, *O. khakicolorata*, *O. parvula*, *O. plicara*, and *O. purpureobrunnea*. Based on a combination of morphology, phylogeny, and holomorphic states, Ou et al. [9] discovered four new species of *Orbilia*, i.e., *O. baisensis*, *O. hanzhongensis*, *O. nanningensis*, and *O. pinea*. In two other studies on plant-associated fungi from Hainan island of China, Liu et al. [10] described three new species of *Microdochium*, i.e., *M. hainanense, M. miscanthi*, and *M. sinense* from plant hosts *Miscanthus sinensis* and *Phragmites australis*; Wang et al. [11] described four new species of *Acrodictys*, namely, *A. bawanglingensis*, *A. diaoluoshanensis*, *A. ellisii,* and *A. pigmentosa,* from dead branches. Focusing on insect pathogenic fungi in Thailand, Thanakitpipattana et al. [12] discovered one new genus, *Neohyperdermium*, and five new species of *Ascopolyporus*, namely, *A. albus* sp. nov., *A. galloides*, *A. griseoperitheciatus*, *A. khaoyaiensis,* and *A. purpuratus*. Moreover, both macroscopic and phylogenetic evidence suggested that *Hyperdermium* is congeneric with *Ascopolyporus* [12]. In arid and semi-arid regions of Northwest China, Zhang et al. [13] discovered and described four new species of Verrucariaceae lichen, namely, *Clavascidium sinense*, *Placidium nigrum*, *Placidium nitidulum*, and *Placidium varium*. From the lichenized fungal genus *Astrothelium*, Jiang et al. [14] described five new species, namely, *A. jiangxiense*, *A. luminothallinum*, *A. pseudocrassum*, *A. subeustominspersum*, and *A. subrufescens*. These excellent works contributed significantly to recognizing fungal species diversity in Ascomycota.

Moving to Basidiomycota, from a residential area of Jiangxi Province in China, Zhang et al. [15] reported an unusual new bryophilous basidiolichen species in the genus *Omphalina*, namely, *O. licheniformis*, providing new insights and evidence for understanding the significance of lichenization during the evolution of Agaricomycetes. From the genus of edible mushrooms *Cantharellus*, Zhang et al. [16] discovered four new species, namely, *C. chrysanthus*, *C. convexus*, *C. neopersicinus,* and *C. sinocinnabarinus*. Demonstrated by both morphological and molecular analysis, Hu et al. [17] discovered eight new species of *Gymnopus* from Northeast China, namely, *G. changbaiensis*, *G. globulosus*, *G. longisterigmaticus*, *G. longus*, *G. macrosporus*, *G. striatus*, *G. tiliicola*, and *G. tomentosus*. Mao et al. [18] described two new species of *Mallocybe* and three new species of *Pseudosperma* from North China, namely, *M. depressa*, *P. gilvum*, *P. laricis*, *M. picea*, and *P. pseudoniveivelatum*. Song et al. [19] revealed the phylogenetic relationships in the genus *Phellodon* (Bankeraceae, Thelephorales) based on multi-locus sequences and described three new species, namely, *P. crassipileatus*, *P. griseofuscus*, and *P. perchocolatus*. Focusing on wood decomposers from a subtropical region of Yunnan Province in China, Luo and Zhao [20] reported three new species of *Trechispora*, namely *T. murina*, *T. odontioidea*, and *T. olivacea*. Kewessa et al. [21] recorded 64 wild edible fungal species belonging to 31 genera and 21 families from the plots established in the natural and plantation forests in Ethiopia, including ecologically and economically important fungal species such as *Agaricus campestroides*, *Tylopilus niger*, *Suillus luteus*, *Tricholoma portentosum*, and *Morchella americana*. The fungal community composition based on sporocarp observation was mainly correlated with the organic matter, available phosphorus, total nitrogen content of the soil, and daily minimum temperature [21].

Beyond the Dikarya and to the early fungal lineage *Conidiobolus* (Ancylistaceae; Entomophthorales; Zoopagomycota), Gryganskyi et al. [22] resolved the phylogeny, lifestyle, and evolution direction of parasitic in *Conidiobolus* group using molecular and genomic data. Gryganskyi et al. [22] found that parasitism evolved multiple times in the Conidiobolus group and suggested that the evolution of ballistic conidia preceded the evolution of the parasitic lifestyle. Focusing on cellular slime molds (Dictyostelids), Cavender et al. [23] discovered four new species, namely, *Cavenderia helicoidea, C. parvibrachiata*, *C. protumula*, and *C. ungulate,* from soil systems in northern Thailand.

Finally, we would like to thank all the contributors to this Special Issue and warmheartedly wish Prof. Wen-Ying Zhuang all the best in the future.
